# CFTR functions as a tumor suppressor in adenoid cystic carcinoma and its silencing reveals an associated vulnerability involving the Hsp70 chaperone system

**DOI:** 10.3389/fonc.2026.1836263

**Published:** 2026-07-06

**Authors:** Gang Zhao, Lixin Li, Qi Zhang, Yucheng Su

**Affiliations:** 1 School of Basic Medicine, Jiamusi University, Jiamusi, China; 2 Stomatology College of Jiamusi University, Jiamusi, China

**Keywords:** adenoid cystic carcinoma, CFTR, HSP (heat shock protein), Hsp70, tumor suppressor

## Abstract

**Introduction:**

Adenoid cystic carcinoma is a rare salivary gland malignancy of the head and neck region characterized by perineural invasion, distant metastasis, and a lack of effective targeted therapies. The molecular mechanisms underlying its progression remain poorly understood.

**Methods:**

In this study, we identified the cystic fibrosis transmembrane conductance regulator (CFTR) as a consistently downregulated gene in adenoid cystic carcinoma through differential expression analysis of multiple independent transcriptomic cohorts. Functional experiments were performed in SACC-83 and SACC-LM cell lines, including ectopic expression and knockdown of CFTR, RNA sequencing, quantitative polymerase chain reaction, and Western blot analyses. Pharmacological inhibition of Hsp70 using VER155008 and activation of the heat shock response by HSF1A were also assessed for effects on cell viability, migration, invasion, and apoptosis. Subcutaneous xenograft experiments in nude mice were conducted to evaluate tumor growth following stable CFTR knockdown in SACC-LM cells.

**Results:**

Preliminary Kaplan-Meier survival analysis demonstrated that low CFTR expression was significantly associated with inferior overall survival, although this finding requires validation in a larger independent cohort. Protein interaction network modeling revealed that CFTR occupies a hub position within a conserved interaction network linking ion channel regulation, protein quality control, and kinase signaling. Functional experiments showed that ectopic expression of CFTR suppressed cell proliferation, migration, and invasion, whereas CFTR knockdown enhanced these malignant phenotypes, supporting a tumor-suppressive role for CFTR in these model systems. RNA sequencing of CFTR-overexpressing cells revealed coordinated downregulation of heat shock protein family members, including HSPA1A, HSPA1B, and HSPA6, with enrichment of pathways related to protein refolding. Quantitative polymerase chain reaction and Western blot analyses confirmed that CFTR expression is inversely associated with Hsp70 family members and MAPK1 (ERK2) at both mRNA and protein levels. Pharmacological inhibition of Hsp70 using VER155008 suppressed cell viability, migration, and invasion in a dose-dependent manner and induced apoptosis, phenocopying the effects of CFTR restoration. Subcutaneous xenograft experiments in nude mice further demonstrated that stable CFTR knockdown in SACC-LM cells markedly accelerated tumor growth. Conversely, activation of the heat shock response by HSF1A promoted proliferation, migration, and invasion, recapitulating the consequences of CFTR loss. Notably, Hsp70 inhibition was accompanied by compensatory upregulation of MAPK1 transcripts, suggesting that CFTR is inversely associated with Hsp70 and MAPK1 through parallel rather than linear mechanisms.

**Discussion:**

These findings indicate that CFTR silencing in adenoid cystic carcinoma is accompanied by elevated Hsp70 chaperone expression, and that pharmacological targeting of Hsp70 phenocopies, rather than necessarily mediates, the tumor-suppressive effects of CFTR restoration in the SACC-83 and SACC-LM lineage. Targeting Hsp70 therefore provides a preclinical rationale, rather than direct translational evidence, for further investigation in this otherwise treatment-refractory malignancy.

## Introduction

Adenoid cystic carcinoma (ACC) is a rare but aggressive malignancy that predominantly originates from the secretory epithelia of the major and minor salivary glands, lacrimal glands, and tracheobronchial submucosal glands (1). Clinically, ACC is characterized by a deceptively indolent initial growth pattern that belies its long-term lethality, a marked predilection for perineural invasion along cranial and peripheral nerve sheaths, and a high propensity for delayed distant metastasis to the lungs, liver, and bone, often manifesting years or even decades after primary treatment (2). Although advances in surgical resection and intensity-modulated radiation therapy have improved locoregional disease control, the long-term prognosis for patients with recurrent or metastatic ACC remains poor, owing largely to the intrinsic chemoresistance of this tumor and the lack of effective molecularly targeted systemic agents (3). At the genomic level, the mutational landscape of ACC is comparatively quiescent relative to other epithelial carcinomas, with tumorigenesis frequently driven by a restricted repertoire of chromosomal rearrangements, most notably the *MYB-NFIB* fusion oncogene, which has been detected in a substantial proportion of cases (4). This relative genomic simplicity, while informative for classification, paradoxically limits the number of actionable therapeutic targets and renders ACC particularly refractory to molecularly guided interventions (5). The identification of novel tumor suppressors and the elucidation of exploitable metabolic or proteostatic vulnerabilities are therefore urgently needed to guide the development of new therapeutic strategies.

The cystic fibrosis transmembrane conductance regulator (*CFTR*), encoded by the *CFTR* gene on chromosome 7q31.2, is a member of the ATP-binding cassette (ABC) transporter superfamily that functions principally as a cAMP-regulated chloride and bicarbonate channel at the apical membranes of secretory and absorptive epithelia (6). While germline loss-of-function mutations in *CFTR* cause cystic fibrosis, the most common lethal autosomal recessive disorder in Caucasian populations, accumulating evidence over the past decade has shown that CFTR plays a more complex and context-dependent role in carcinogenesis than previously appreciated (7). In certain malignancies, CFTR has been reported to exhibit oncogenic properties; however, in a growing number of epithelial cancers, particularly those arising from intestinal, respiratory, and gastric tissues, CFTR has been recognized as a tumor suppressor whose epigenetic silencing or transcriptional downregulation promotes malignant transformation and disease progression (8). Loss of CFTR function has been linked to widespread disruption of cellular homeostasis, including dysregulation of intracellular pH and transmembrane ion gradients, aberrant activation of Wnt-β-catenin signaling, and constitutive engagement of NF-κB-mediated inflammatory pathways, collectively creating a cellular environment permissive for tumor initiation and sustained neoplastic growth (9). Epidemiological data further indicate that patients with cystic fibrosis carry a significantly elevated risk of gastrointestinal malignancies, providing independent clinical support for the tumor-suppressive role of CFTR in epithelial tissues (10). Yet despite the physiological relevance of CFTR to the secretory glandular epithelia from which ACC originates, its expression, prognostic significance, and biological function in this malignancy remain unexplored. Importantly, salivary gland acinar and ductal cells depend on CFTR-mediated chloride and bicarbonate secretion for primary fluid production and luminal pH regulation. Because ACC is thought to arise from the intercalated duct and myoepithelial compartments of these secretory epithelia, the transcriptional silencing of CFTR would be predicted to disrupt the differentiated secretory phenotype of the tissue of origin. Furthermore, the constitutive secretory activity of salivary gland cells imposes substantial demand on the endoplasmic reticulum proteostasis machinery, a circumstance that may render these cells particularly sensitive to perturbations in CFTR-associated protein quality control. These tissue-specific considerations provide a biological context for investigating CFTR function in ACC that extends beyond the gastrointestinal and respiratory epithelial paradigms in which CFTR has been most extensively studied.

Beyond its role as an ion channel, CFTR is a large, polytopic transmembrane glycoprotein of 1480 amino acid residues whose complex multi-domain structure imposes a substantial folding burden on the endoplasmic reticulum (ER) (11). CFTR biogenesis is inherently inefficient even under physiological conditions, with a significant fraction of newly synthesized wild-type protein failing to achieve native conformation and instead being targeted for ER-associated degradation via the ubiquitin-proteasome pathway (12). Maturation of CFTR is therefore strictly dependent on the cellular proteostasis network, particularly the heat shock protein 70 (HSP70) chaperone system, including HSPA1A and its co-chaperones of the DNAJ/HSP40 family, which together facilitate recognition, assisted folding, and quality control of nascent CFTR polypeptides at the ER membrane (13). Because its error-prone folding process constitutes a continuous source of proteotoxic burden, CFTR biosynthesis is intrinsically coupled to the activation of cellular stress responses (14). CFTR also participates in the assembly of macromolecular signaling complexes that coordinate cAMP-dependent protein kinase (PKA) activity at the plasma membrane, serving not only as a substrate of PKA-mediated phosphorylation but also as an upstream modulator of a signaling axis that can inhibit cell cycle progression and proliferation in specific cellular contexts (15). These observations raise the possibility that the transcriptional silencing of CFTR in ACC may have functional consequences extending beyond the loss of ion channel activity, potentially affecting both proteostasis and growth-regulatory signaling. However, whether and how these aspects of CFTR biology contribute to ACC pathogenesis has not been previously investigated.

In the present study, we integrated multi-cohort transcriptomic analysis with experimental validation to characterize the role of CFTR in ACC. Analysis of independent GEO datasets identified CFTR as a consistently downregulated gene in tumor tissues, and Kaplan-Meier analysis revealed that reduced CFTR expression is associated with inferior patient survival. CFTR was prioritized for functional investigation based on three converging lines of evidence: first, CFTR exhibited consistently large-magnitude downregulation across both microarray and RNA-seq platforms in the primary discovery cohorts; second, among the core downregulated genes, CFTR demonstrated the most significant prognostic association with overall survival; and third, CFTR occupied a central hub position in the protein-protein interaction network connecting ion transport, proteostasis, and kinase signaling modules. This multi-layered convergence distinguished CFTR from other deregulated genes that met only one or two of these prioritization criteria. Gene set enrichment analyses revealed that CFTR-high tumors were enriched for secretory and cAMP-related pathways, whereas CFTR-low tumors exhibited enrichment of ribosome biogenesis and spliceosome pathways, suggesting a shift in transcriptional programs associated with CFTR loss. Using SACC-LM and SACC-83 cell lines, we showed that restoring CFTR expression suppresses proliferation, migration, and invasion, and that stable CFTR re-expression is accompanied by downregulation of Hsp70 family chaperones and MAPK1 at both the mRNA and protein levels. These findings support a tumor-suppressive role for CFTR in ACC and reveal an association between CFTR loss and increased cellular reliance on the Hsp70 chaperone network. Pharmacological inhibition of Hsp70 phenocopied the suppressive effects of CFTR restoration *in vitro*, suggesting that the proteostatic vulnerability associated with CFTR deficiency may represent a potential therapeutic opportunity warranting further preclinical evaluation.

## Methods

### Acquisition and preprocessing of public transcriptomic datasets

Gene expression profiles for adenoid cystic carcinoma were obtained from the Gene Expression Omnibus database. Datasets GSE36820 (Affymetrix microarray; ACC tumor vs normal salivary gland), GSE153230 (RNA-seq; ACC tumor vs paired non-tumor tissue), and GSE214969 (Affymetrix Clariom S Assay, 46 ACC tumor samples) were included for primary analysis and validation (16–18). Platform-specific normalization was applied as follows: Affymetrix microarray data (GSE36820) were normalized using robust multi-array average (RMA); RNA-seq count data were normalized using the trimmed mean of M-values (TMM) method followed by voom transformation for limma-based analysis, and variance-stabilizing transformation (VST) in DESeq2-based analysis. Each dataset was processed independently without cross-dataset merging. Within each dataset, the ComBat algorithm (sva R package) was applied to correct for internal technical batch variation among samples when annotated batch information was available. No cross-platform expression matrix was generated, and no cross-dataset ComBat correction was performed; each dataset was analyzed entirely independently. Differential expression analysis was then performed separately within each dataset to ensure that the downregulation of CFTR and other genes could be validated independently across cohorts. The survival cohort comprised all patients with both CFTR expression data and available overall-survival follow-up in the relevant cohort; samples lacking survival annotation were excluded. The optimal cutoff threshold was set at no less than 25%, resulting in a High Group of 21 samples (72.41%) and a Low Group of 8 samples (27.59%).

### Differential gene expression and survival analysis

Differential expression analysis was conducted as follows: DESeq2 (with Wald test) was applied to the RNA-seq dataset GSE153230, given its count-based statistical framework; limma was applied to the microarray dataset GSE36820, consistent with best practices for array-based data. Genes with an absolute log2 fold change greater than 1 and an adjusted P value less than 0.05 were considered significant. The dataset GSE214969 was used exclusively for independent validation of expression patterns and pathway enrichment rather than for differential expression testing. GSE153283 was processed independently using the same analytical pipeline as the primary datasets to verify the broader transcriptional landscape. For the core signature, genes meeting the significance threshold of absolute log2 fold change greater than 1 and adjusted P value less than 0.05 in both the DESeq2 analysis of GSE153230 and the limma analysis of GSE36820 were identified by intersection as the commonly downregulated set. An intersection-based strategy was selected to identify genes with consistent suppression across both platform types, prioritizing reproducibility over sensitivity. The core downregulated genes were annotated using the Ensembl BioMart database (19). Expression consistency was validated across datasets using clustered heatmaps. The prognostic value of CFTR expression was assessed via Kaplan-Meier survival analysis and the log-rank test. An additional dataset GSE153283 was processed similarly to further validate the transcriptional landscape. The optimal cut-point stratifying patients into CFTR-high and CFTR-low groups was determined using the maximally selected rank statistic implemented in the “surv_cutpoint” function of the “survminer” R package (based on the “maxstat” method), with a minimum group-proportion constraint of ≥25% to avoid extreme imbalance. Applying this criterion, the survival cohort (n = 29 patients with both CFTR expression data and available overall-survival follow-up) was stratified into a CFTR-high group (n = 21, 72.41%) and a CFTR-low group (n = 8, 27.59%); during the follow-up period, 20 death events were recorded among the 29 patients, with 9 patients remaining alive at the last follow-up. A formal multivariable Cox proportional-hazards analysis was not feasible because clinical covariates such as TNM stage, perineural invasion and adjuvant therapy were not uniformly annotated in the public cohort. All analyses were performed in R.

### Protein network construction and functional enrichment analysis

Protein-protein interaction networks for the core downregulated genes were constructed using the STRING database with a medium confidence score threshold of 0.400 (20). The network was visualized in Cytoscape and hub genes were identified based on network topology parameters (21). Functional annotation of CFTR and its interactors was performed using the STRING enrichment module encompassing Gene Ontology and Reactome pathway analyses. Gene Set Enrichment Analysis was conducted on the GSE214969 (GPL23159, 46 ACC tumor samples) dataset to evaluate pathway differences based on CFTR expression levels using Kyoto Encyclopedia of Genes and Genomes pathways.

### Transcriptomic profiling by RNA sequencing

A CFTR overexpression model was established in an adenoid cystic carcinoma cell line via transient transfection. Total RNA was extracted and libraries were prepared for paired-end sequencing on an Illumina NovaSeq 6000 platform. Raw reads were quality-filtered and aligned to the GRCh38 reference genome using HISAT2 (22). Gene counts were quantified with featureCounts and differential expression analysis was performed using DESeq2. Genes with an adjusted P value less than 0.05 and an absolute log2 fold change greater than 0.3 were considered differentially expressed. Significantly downregulated genes were subjected to Gene Ontology and Kyoto Encyclopedia of Genes and Genomes pathway enrichment analyses using the clusterProfiler package (23). A protein-protein interaction network of significant genes was constructed via STRING.

### Cell culture and functional assays

Salivary adenoid cystic carcinoma cell lines SACC-83 and SACC-LM were cultured in Dulbecco’s Modified Eagle Medium supplemented with 10% fetal bovine serum and 1% penicillin–streptomycin at 37 °C in a humidified atmosphere with 5% CO2. SACC−83 (catalog No. FH0798) and SACC−LM (catalog No. FH0799) were obtained from Fuheng Biology (https://www.fudancell.com/), Shanghai, China. The identity of SACC-83 and SACC-LM cell lines was authenticated by short tandem repeat (STR) profiling, and both lines were confirmed to be free of mycoplasma contamination. Cells were seeded at a density of 2 times 10 to the power of 5 cells per well in 6-well plates and allowed to attach overnight. Transient transfection was performed at approximately 70 to 80 percent confluence using Lipofectamine 3000 reagent (Thermo Fisher Scientific, L3000001). For each well, 2.5 micrograms of plasmid DNA (CFTR overexpression construct or empty vector) and 5 microliters of P3000 reagent were diluted in 125 microliters of Opti-MEM reduced serum medium, and 5 microliters of Lipofectamine 3000 was diluted separately in 125 microliters of Opti-MEM. The two solutions were combined, incubated for 15 minutes at room temperature, and added to cells. The medium was replaced with complete growth medium 6 hours after transfection. For Hsp70 knockdown and overexpression, Hsp70-targeting shRNA or Hsp70 expression constructs were transfected using the same Lipofectamine 3000 protocol. For shRNA knockdown, a CFTR-targeting shRNA construct (5′-GTGATTCTTTCGACCAATTTA-3′) or a non-targeting scrambled shRNA control was transfected using the same protocol. The efficiency of CFTR overexpression and knockdown was independently verified in both SACC-83 and SACC-LM cells at the transcript and protein levels by RT-qPCR and Western blot prior to downstream functional assays. For stable lines, transfected cells were selected with puromycin (2 μg/mL) for 14 days as appropriate. The efficiency of CFTR overexpression and knockdown was independently verified in both SACC-83 and SACC-LM cells at the transcript and protein levels by RT-qPCR and Western blot (**Supplementary Figure 1**) prior to downstream functional assays. Cell migration was evaluated by wound healing assay in which confluent monolayers were scratched with a sterile 200-μL pipette tip and photographed at 0 h and 24 h. Cell invasion was evaluated using Matrigel-coated Transwell chambers (Corning, 3422; Matrigel, Corning, 356234) respectively. Cell proliferation was monitored over 96 hours using the Cell Counting Kit-8 assay.

### Quantitative real-time polymerase chain reaction and Western blot

Total RNA was extracted using TRIzol reagent (Invitrogen, 15596026CN) and reverse-transcribed into cDNA. Quantitative real-time polymerase chain reaction was performed using SYBR Green One-Step qRT-PCR Kit (BeyoFast, D7268S) to measure the expression of HSPA1A, HSPA1B, HSPA6, and MAPK1 with GAPDH as the internal reference. One-step qRT-PCR was performed as follows: reverse transcription at 50 °C for 15 min, followed by pre-denaturation at 95 °C for 2 min. The thermal cycling program consisted of 40 cycles of 95 °C for 15 sec and 60 °C for 15–30 sec, with melt curve analysis (95 °C for 15 sec, 60 °C for 15 sec, 95 °C for 15 sec). Relative gene expression was calculated using the 2 to the power of negative delta delta Ct method. The primer sequences (5′–3′) were as follows: HSPA1A forward, ATCCAGTGTTCCGTTTCCA; reverse, TCAAAGATGAGCACGTTGC. HSPA1B forward, CAAGGCCAACAAGATCACC; reverse, CTCGATCTCCTCCTTGCTC. HSPA6 forward, TTTCACCACCTACTCGGAC; reverse, CCTCTCACCCTCATACACC. MAPK1 forward, GATCTCAAGATCTGTGACTTTGG; reverse, CACATATTCTGTCAGGAACCC. For protein analysis cells were lysed in radioimmunoprecipitation assay buffer (Beyotime, P0013B) containing protease and phosphatase inhibitors (Beyotime, P1045). Protein concentration was determined using the BeyoBCA Rapid Protein Assay Kit (Beyotime, P0398M). Equal amounts of protein were separated by 10 percent SDS-PAGE. Proteins were transferred to polyvinylidene fluoride membranes (Millipore, IPVH00010). Membranes were blocked with 5 percent non-fat milk in TBST for 1 hour at room temperature and then incubated overnight at 4 degrees Celsius with primary antibodies diluted in blocking buffer. Following three washes with TBST, membranes were incubated with secondary antibody (CST, Cat# 7074, 1:5000) for 1 hour at room temperature and detected using enhanced chemiluminescence substrate (Beyotime, P0018M). The primary antibodies were obtained from Cell Signaling Technology (CST): Hsp70 (Cat# 4872, 1:1000), GAPDH (Cat# 2118,1:2000), and MAPK1 (Cat# 9108,1:1000).

### Pharmacological treatment and apoptosis analysis

The functional impact of Hsp70 modulation was assessed using the inhibitor VER155008 (MedChemExpress, HY10941) and the heat shock response activator HSF1A (MedChemExpress, HY103000). Both compounds were dissolved in DMSO, and the final DMSO concentration in all treatment conditions, including vehicle controls, did not exceed 0.1 percent. For migration and invasion assays, cells were treated with VER155008 (40 μM) or HSF1A (1500 nM) for 24 h. For apoptosis analysis, cells were treated with VER155008 (40 μM) or HSF1A (1500 nM) for 24 h. SACC-83 and SACC-LM cells were treated with the indicated concentrations of VER155008 (0, 10, 20, 40 μM) or HSF1A (0, 200, 500, 1000, 1500 nM) for 24 h for viability dose–response analysis. Cell viability was measured using the Cell Counting Kit-8 assay (Beyotime, C0038). Apoptosis was quantified by dual-fluorescence flow cytometry using the Annexin V-FITC/PI Apoptosis Detection Kit (Beyotime, C1062M). Flow cytometric gating was performed as follows: doublets were excluded based on FSC-A versus FSC-H; the live-cell gate was defined from unstained and single-stained compensation controls; Annexin V-FITC and PI quadrant gates were set using unstained, single-stained and untreated controls to distinguish viable (Annexin V−/PI−), early apoptotic (Annexin V+/PI−), late apoptotic (Annexin V+/PI+) and necrotic (Annexin V−/PI+) populations.

### Animal experiments

All animal procedures were performed in accordance with institutional guidelines and approved by the Institutional Animal Care and Use Committee. Female BALB/c nude mice (4–6 weeks old) were purchased from Liaoning Changsheng Biotechnology CO., LTD (https://www.lncssw.com/) and housed under specific-pathogen-free conditions with 12-h light/dark cycles and free access to food and water. SACC-LM cells stably transfected with an shRNA construct targeting CFTR (shCFTR) or a non-targeting shNC were harvested, resuspended in serum-free medium and inoculated subcutaneously into the right flank (5 × 10^6^ cells per mouse; n = 6 per group). Tumor dimensions were measured every 3 days with vernier calipers from the time tumors became palpable, and tumor volume was calculated as V = 0.5 × length × width^2^. Animals were humanely euthanized when tumors reached the pre-defined humane end-point, and tumors were excised, photographed and weighed. All animal experiments (subcutaneous xenograft models using BALB/c nude mice) were approved by the Biomedical and Medical Ethics Committee of the School of Basic Medical Sciences, Jiamusi University (approval No. JDJCYXY20260019). All procedures complied with the ARRIVE guidelines and relevant national regulations”.

### Statistical analysis

All experimental assays were performed as at least three independent biological replicates, each with technical triplicates unless stated otherwise. Data are presented as mean ± standard deviation (SD) unless otherwise indicated in the figure legends. Normality of data distribution was assessed using the Shapiro–Wilk test. Two-group comparisons were analysed using the unpaired two-tailed Student’s t-test when data conformed to normality and equal variance, and the Mann–Whitney U test otherwise. Multiple-group comparisons were performed by one-way ANOVA followed by Tukey’s or Dunnett’s *post-hoc* test, as appropriate; dose–response CCK-8 and time-course assays were analysed by two-way ANOVA with Šidák’s correction for multiple comparisons. A P value less than 0.05 was considered statistically significant. All statistical analyses were performed using GraphPad Prism 8 and R 4.3.

## Results

### CFTR is consistently downregulated in ACC and its reduced expression is associated with poor patient survival

**Figure 1:**
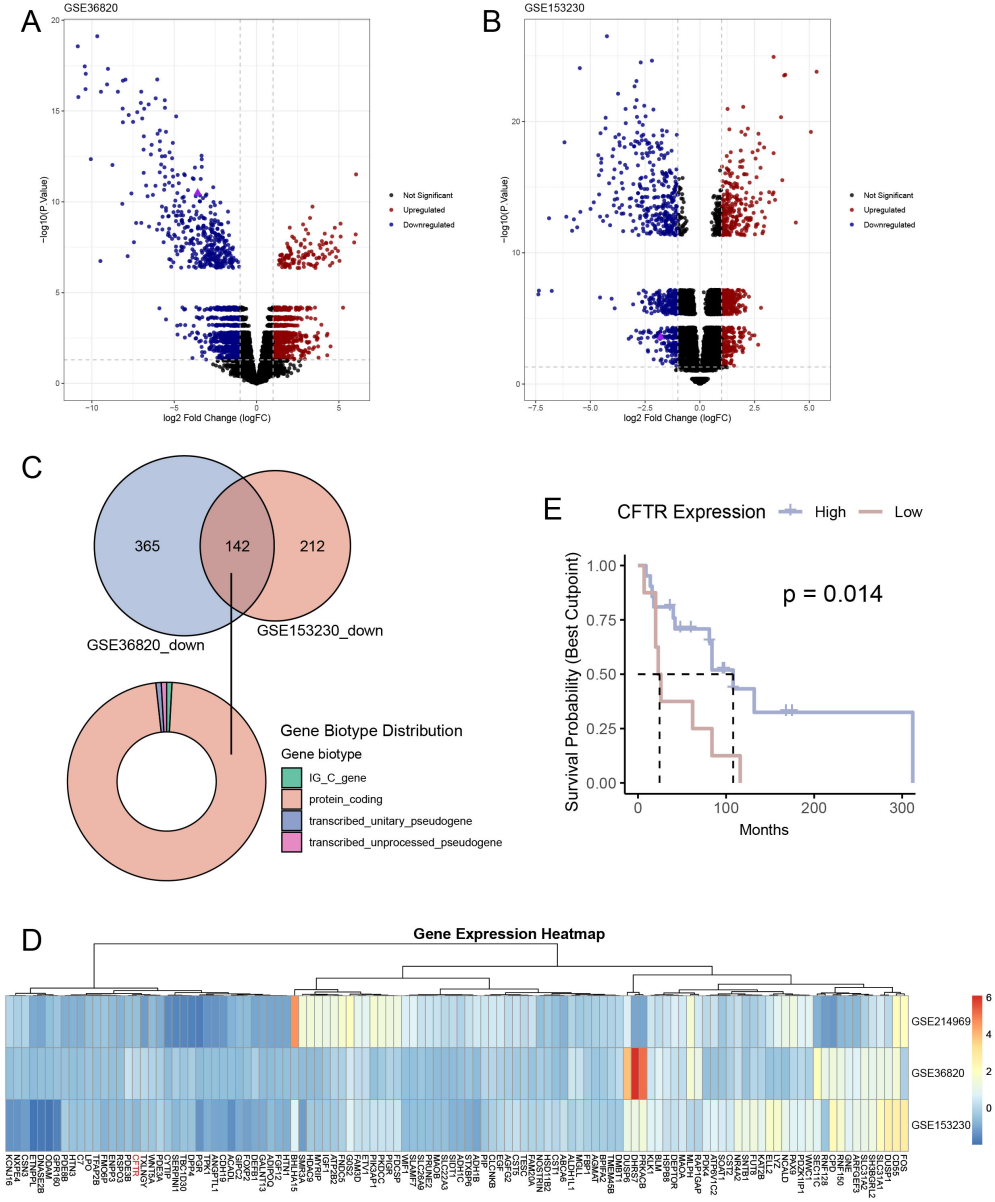
CFTR is significantly downregulated in adenoid cystic carcinoma and its low expression is associated with inferior overall survival in a public transcriptomic cohort. **(A)** Volcano plot of differentially expressed genes between tumor and normal tissues in the GSE36820 dataset, with CFTR indicated by a purple triangle in the significantly downregulated region. **(B)** Volcano plot of differentially expressed genes between tumor and normal tissues in the GSE153230 dataset, with CFTR similarly marked in the significantly downregulated region. **(C)** Venn diagram (upper panel) showing the intersection of significantly downregulated genes between GSE36820 and GSE153230, yielding 142 commonly downregulated genes; donut chart (lower panel) illustrating the gene biotype distribution of the 142 overlapping genes, with protein-coding genes constituting the predominant category. **(D)** Heatmap displaying the expression patterns of the 142 core downregulated genes across three independent datasets (GSE214969, GSE36820, and GSE153230), where blue denotes low expression and red denotes high expression, with hierarchical clustering applied to both rows and columns. **(E)** Kaplan-Meier survival curves for ACC patients stratified by CFTR expression level using the optimal cutpoint (Best Cutoff Value = 5.5688), with the blue line representing the high-expression group(n = 21, 72.41%) and the brown line representing the low-expression group(n = 8, 27.59%); the P value was determined by the log-rank test.

We performed differential expression analysis on datasets GSE36820 and GSE153230 to identify recurrent transcriptional alterations in adenoid cystic carcinoma. Volcano plots showed that *CFTR* ranked among the most significantly downregulated genes in both cohorts with large negative log2 fold changes and significant adjusted P values (**Figures 1A, B**). Both datasets exhibited numerous gene expression changes between tumor and normal tissues. *CFTR* consistently occupied the lower-left quadrant in these plots, confirming its marked transcriptional suppression in adenoid cystic carcinoma independent of the profiling platform.

Intersecting the significantly downregulated gene lists from both datasets yielded 142 commonly suppressed genes, compared to 365 unique to GSE36820 and 212 unique to GSE153230 (**Figure 1C**). *CFTR* was among these shared genes, indicating its transcriptional loss is a recurrent molecular feature of the disease. Biotype annotation showed the majority of these genes were protein-coding, alongside minor contributions from pseudogenes and immunoglobulin C genes (**Figure 1C**). This protein-coding predominance suggests the downregulation reflects functionally relevant transcriptional reprogramming rather than stochastic changes in non-coding loci.

We examined the expression of these 142 genes across three independent datasets, including the validation cohort GSE214969, to assess the generalizability of this core gene set. Hierarchical clustering demonstrated broad suppression of these genes in tumor samples across all cohorts (**Figure 1D**). While a few genes showed elevated expression in isolated samples due to inter-tumor heterogeneity, the overall trend of coordinated downregulation remained consistent. This cross-cohort reproducibility confirms that these genes define a conserved transcriptional signature of adenoid cystic carcinoma.

We evaluated the prognostic significance of *CFTR* expression using Kaplan-Meier analysis. Patients with low *CFTR* expression experienced significantly shorter overall survival compared to the high-expression group (**Figure 1E**). The survival curves diverged early, and the low-expression group showed a steep initial decline with a notably shorter median survival time. This separation persisted throughout the 300-month observation period, indicating that *CFTR* status reflects a durable biological distinction in disease progression.

These findings establish CFTR as a consistently downregulated gene in adenoid cystic carcinoma across multiple independent datasets and suggest an association between its reduced expression and inferior patient survival. The survival analysis is based on a cohort of 29 patients and should be considered preliminary. Confirmation in a larger independent cohort with multivariable adjustment for established prognostic factors is required. The identification of CFTR within a broader program of coordinated gene suppression and its potential prognostic relevance warrant further investigation of its functional role in this malignancy.

### CFTR occupies a central hub position within a conserved interaction network and its loss is associated with activation of proliferative and ribosomal pathways

We delineated the functional consequences of *CFTR* loss and its associated signaling networks through differential expression profiling, protein network modeling, gene set enrichment, and pathway annotation.

**Figure 2:**
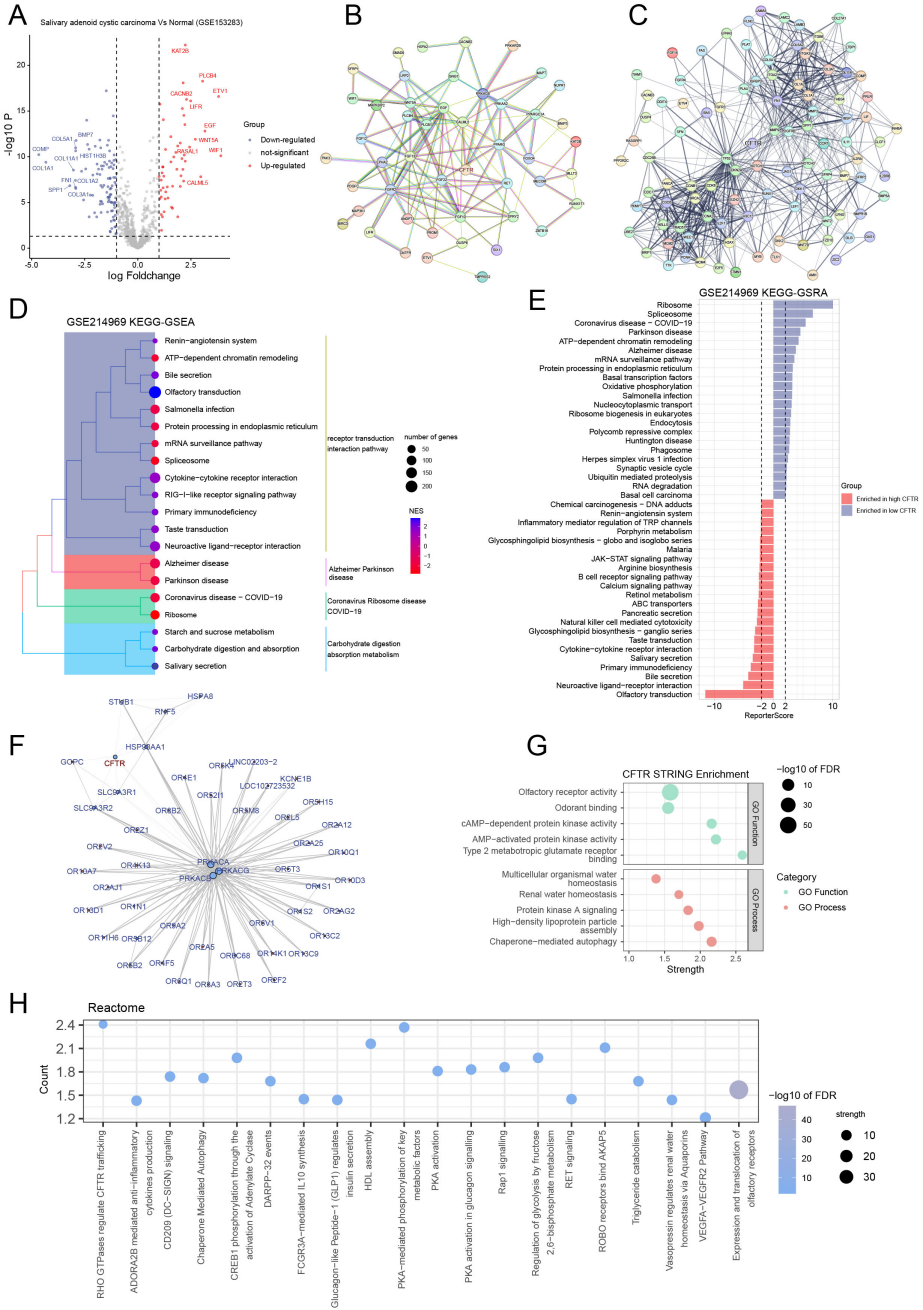
Bioinformatics characterization of downstream signaling pathways and protein interaction networks associated with *CFTR* in adenoid cystic carcinoma. **(A)** Volcano plot of differentially expressed genes between salivary adenoid cystic carcinoma and normal tissues in the GSE153283 dataset, with red dots indicating significantly upregulated genes, blue dots indicating significantly downregulated genes, and gray dots representing genes without statistical significance. **(B)** Protein-protein interaction (PPI) network of the 142 core downregulated genes constructed using the STRING database, where nodes represent proteins and edges represent interactions. **(C)** Expanded PPI network incorporating additional known interacting proteins, with CFTR occupying a highly connected hub position. **(D)** Bubble plot of Gene Set Enrichment Analysis (GSEA) results based on KEGG pathways in the GSE214969 dataset stratified by *CFTR* expression, where bubble size indicates the number of genes in each pathway and color reflects the normalized enrichment score, with red denoting enrichment in the high *CFTR* group and blue denoting enrichment in the low *CFTR* group. **(E)** Bidirectional bar chart of Gene Set Regulatory Analysis results for KEGG pathways in the GSE214969 dataset, with the x-axis representing the ReporterScore; red bars indicate pathways enriched in the high *CFTR* expression group and blue bars indicate pathways enriched in the low *CFTR* expression group. **(F)** Focused sub-network centered on CFTR, highlighting direct interactions with key signaling molecules including PRKACA and PRKACB. **(G)** Bubble plot of Gene Ontology enrichment analysis for CFTR and its interacting proteins, with the x-axis representing enrichment strength, bubble size corresponding to the negative log10 of the false discovery rate, and colors distinguishing Molecular Function from Biological Process categories. **(H)** Bubble plot of Reactome pathway enrichment analysis for CFTR and its interacting proteins, with the x-axis listing individual pathway terms, the y-axis indicating gene count, bubble size representing enrichment strength, and color intensity reflecting the negative log10 of the false discovery rate.

Analysis of the independent dataset GSE153283 confirmed broad transcriptional reprogramming in adenoid cystic carcinoma (**Figure 2A**). The volcano plot showed *KAT2B*, *ETV1*, *PLCB4*, and *CACNB2* among the most significantly upregulated genes, while extracellular matrix components *COL1A1*, *COL5A1*, *COL11A1*, and *FN1* were highly downregulated. This landscape is consistent with the primary datasets, reinforcing that the disease features broad suppression of tissue homeostasis genes alongside aberrant oncogenic activation.

Protein network construction from the 142 core downregulated genes revealed a densely interconnected architecture (**Figure 2B**). *CFTR* emerged as a highly connected node interacting with numerous downregulated proteins. Expanding the network to include known proteome interactors further established its hub status (**Figure 2C**). Sub-network analysis identified a prominent interaction axis between *CFTR* and the catalytic subunits of protein kinase A, *PRKACA* and *PRKACB* (**Figure 2F**). Additional interactors included *GOPC*, solute carrier family members, heat shock proteins, *STMB1*, and *RNF5*. This suggests *CFTR* participates in a hub connecting ion channel regulation, protein quality control, and kinase signaling. Olfactory receptor family members at the network periphery may share upstream regulatory elements with CFTR, although the specific signaling mediators remain to be determined.

Gene set enrichment analysis on the GSE214969 cohort stratified by *CFTR* expression yielded biologically coherent patterns (**Figure 2D**). Pathways enriched in the *CFTR*-high group included olfactory transduction, bile secretion, neuroactive ligand-receptor interaction, taste transduction, salivary secretion, and the renin-angiotensin system. This aligns with the role of *CFTR* as an epithelial chloride channel and suggests residual *CFTR* preserves the normal secretory phenotype. Pathways enriched in the *CFTR*-low group included ribosome biogenesis, spliceosome, and ATP-dependent chromatin remodeling. The ribosomal and spliceosomal enrichment indicates that *CFTR* loss couples with a metabolic shift toward heightened translational capacity, a hallmark of rapidly proliferating cancers. Gene set regulatory analysis corroborated these findings by assigning the highest positive scores to secretory pathways and the strongest negative scores to ribosome, spliceosome, and oxidative phosphorylation pathways (**Figure 2E**).

Functional enrichment of *CFTR* and its direct interactors provided further mechanistic resolution (**Figure 2G**). Enriched molecular function terms included cyclic AMP-dependent protein kinase activity and AMP-activated protein kinase activity. Biological process terms encompassed multicellular organismal water homeostasis, protein kinase A signaling, and chaperone-mediated autophagy. The convergence on cyclic AMP-dependent kinase activity and protein kinase A signaling in the enrichment analysis suggests a potential functional association between CFTR and the protein kinase A pathway, which requires direct experimental validation. Reactome enrichment identified pathways involving RHO GTPases, protein kinase A activation, and chaperone-mediated autophagy (**Figure 2H**). Because the connection between CFTR and the cAMP and PKA axis is derived exclusively from protein interaction databases and pathway enrichment, it should be regarded as a bioinformatically generated hypothesis rather than an established mechanism in ACC.

These analyses suggest that CFTR expression is associated with the maintenance of a differentiated epithelial secretory phenotype. Bioinformatic enrichment implicates cyclic AMP and protein kinase A-related pathways in this association, although no direct biochemical measurement of cAMP levels or PKA activity was performed. CFTR-low tumors exhibited enrichment of ribosome biogenesis and spliceosome pathways, indicating a shift toward a translationally hyperactive state. The enrichment of chaperone-mediated autophagy suggests CFTR and its interactors participate in protein homeostasis pathways whose dysregulation may promote tumor survival under proteotoxic stress.

### CFTR overexpression is associated with reduced heat shock protein expression and endoplasmic reticulum stress

**Figure 3:**
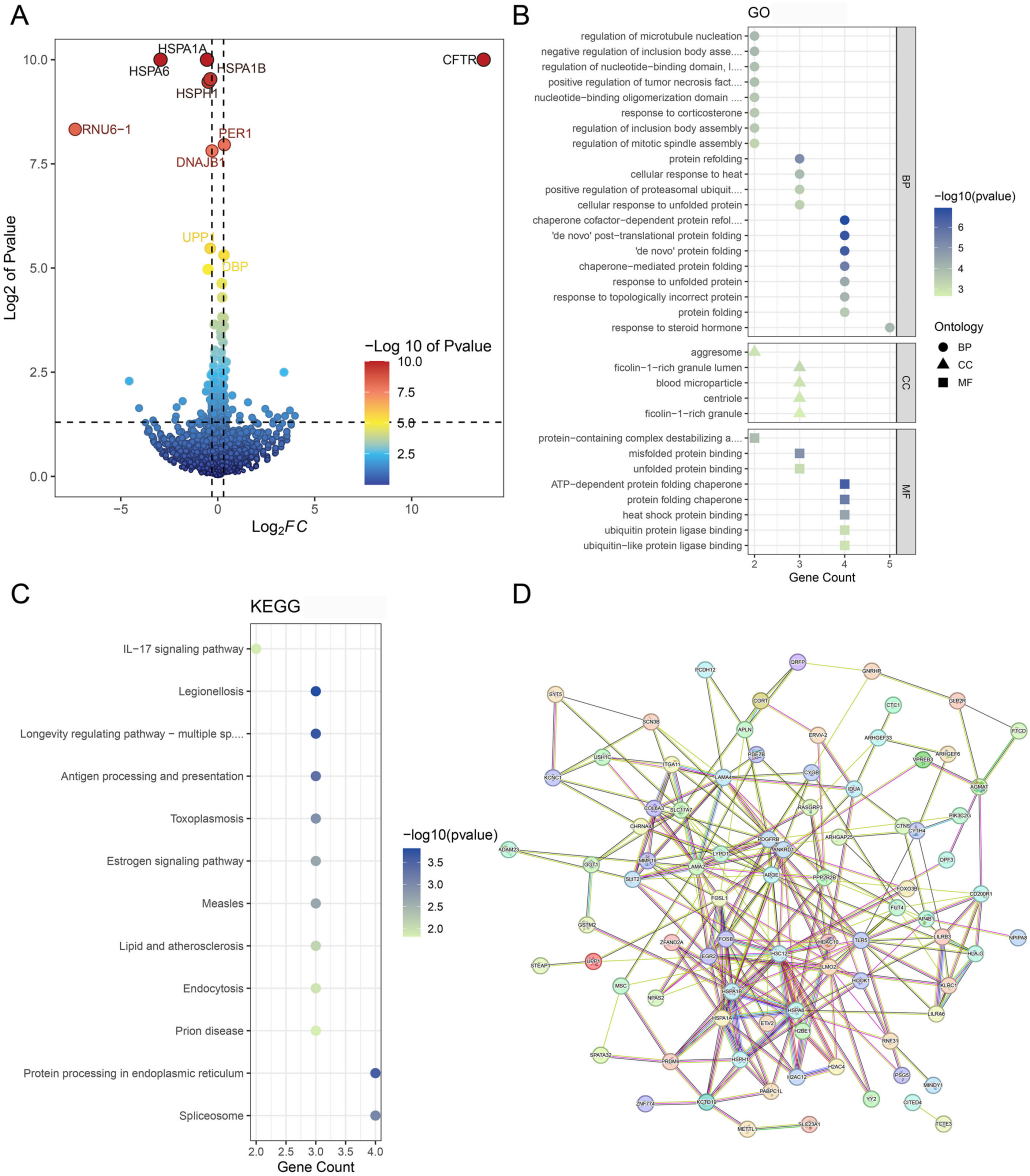
CFTR overexpression is associated with reduced heat shock protein expression and proteostasis-related transcriptional changes in SACC-83 cells. **(A)** Volcano plot of differentially expressed genes between CFTR-overexpressing and control ACC cells, with the x-axis representing log2 fold change and the y-axis representing log2-transformed P value. **(B)** Gene Ontology enrichment analysis of downregulated differentially expressed genes, organized into Biological Process (circles), Cellular Component (triangles), and Molecular Function (squares) categories, with the x-axis indicating gene count and color intensity representing the negative log10-transformed P value. **(C)** KEGG pathway enrichment analysis dot plot of downregulated differentially expressed genes, with the x-axis indicating gene count and color intensity representing the negative log10-transformed P value; **(D)** The protein interaction network of all-regulated genes.

We performed RNA sequencing on adenoid cystic carcinoma cells following transient CFTR overexpression to investigate the transcriptional consequences of CFTR restoration. The volcano plot confirmed successful transfection, with CFTR showing the largest positive log2 fold change (**Figure 3A**). Notably, heat shock protein family members were coordinately downregulated. HSPA1A, HSPA1B, HSPA6, HSPH1, and DNAJB1 ranked among the most significantly suppressed genes, with HSPA1A displaying particularly robust downregulation. This selective chaperone downregulation suggests that CFTR restoration is accompanied by reduced proteostatic stress in these cells.

Gene Ontology enrichment of the downregulated genes centered on proteostasis and stress responses (**Figure 3B**). Top biological process terms included protein refolding, chaperone-mediated protein folding, and cellular responses to unfolded proteins and heat. Cellular component terms included the aggresome and stress granules, while molecular function terms involved unfolded protein binding and heat shock protein binding. Pathway enrichment corroborated these results, with protein processing in the endoplasmic reticulum emerging as the most significant pathway (**Figure 3C**).

The protein interaction network of all-regulated genes was chaperone-dominated (**Figure 3D**). A dense core of heat shock proteins, including HSPA1A, HSPA1B, HSPA6, and HSP90AA1, connected to ubiquitin-proteasome components, vesicular trafficking machinery, and cytoskeletal regulators. The coordinated suppression of this network implies that adenoid cystic carcinoma cells maintain elevated basal proteostatic stress in the absence of functional CFTR.

### CFTR restoration attenuates the malignant phenotypes of adenoid cystic carcinoma cells *in vitro* and *in vivo*

**Figure 4:**
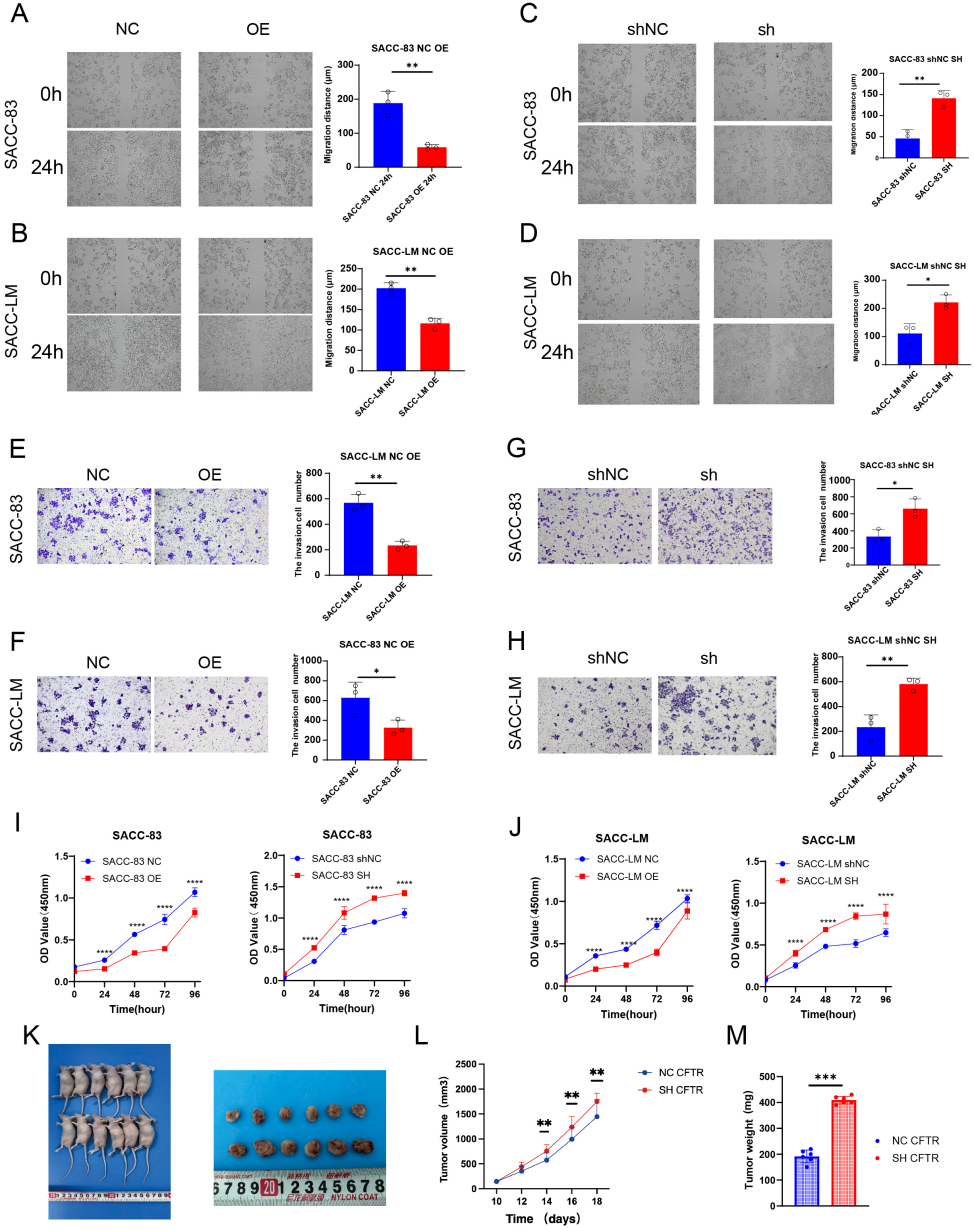
Modulation of CFTR expression alters the migration, invasion, and proliferation of SACC-83 and SACC-LM cells *in vitro*, and CFTR knockdown accelerates xenograft tumor growth *in vivo*. **(A, B)** Representative phase-contrast images and quantification of wound-healing assays in SACC-83 **(A)** and SACC-LM **(B)** cells transfected with overexpression construct (OE) or negative control (NC), captured at 0 h and 24 h after scratch generation; the y-axis represents migration distance (μm). **(C, D)** Representative phase-contrast images and quantification of wound-healing assays in SACC-83 **(C)** and SACC-LM **(D)** cells transfected with short-hairpin RNA (sh) or non-targeting control (shNC), captured at 0 h and 24 h. **(E, F)** Representative crystal-violet-stained photomicrographs and quantification of Transwell invasion assays in SACC-83 **(E)** and SACC-LM **(F)** cells comparing OE and NC groups; the y-axis represents the number of invaded cells. **(G, H)** Representative photomicrographs and quantification of Transwell invasion assays in SACC-83 **(G)** and SACC-LM **(H)** cells comparing sh and shNC groups. **(I)** CCK-8 proliferation curves for SACC-83 cells, with the left subpanel comparing NC and OE groups and the right subpanel comparing shNC and sh groups; the x-axis represents time (0, 24, 48, 72, and 96 h) and the y-axis represents the OD value at 450 nm. **(J)** CCK-8 proliferation curves for SACC-LM cells, arranged as in **(I)**. Representative image of three independent biological replicates; densitometric quantification is derived from n = 3 biologically independent experiments. **(K)** Representative photographs of BALB/c nude mice bearing subcutaneous xenografts (left) and excised end-point tumors (right) from the two groups (n = 6 per group). **(L)** Tumor growth curves of subcutaneous xenografts in BALB/c nude mice inoculated with cells stably transfected with negative control (NC CFTR) or CFTR-knockdown (SH CFTR) constructs (n = 6); tumor volume was measured every 2 days. **(M)** End-point tumor weights. Tumor growth curves were compared by two-way ANOVA and end-point tumor weights by unpaired two-tailed Student’s t-test. *P < 0.05, **P < 0.01, ***P < 0.001, ****P < 0.0001.

To investigate the biological function of the cystic fibrosis transmembrane conductance regulator (CFTR) in adenoid cystic carcinoma, we evaluated the effects of CFTR overexpression and knockdown on cell migration, invasion, and proliferation in SACC-83 and SACC-LM cell lines. The wound healing assay revealed that ectopic expression of CFTR significantly reduced the migration distance of SACC-83 and SACC-LM cells compared to the negative control group (**Figures 4A, B**). Conversely, the depletion of endogenous CFTR markedly accelerated cell migration in both cell lines (**Figures 4C, D**). We further assessed the invasive capacity of these cells using Transwell assays. Consistent with the migration results, CFTR overexpression dramatically decreased the number of invading cells (**Figures 4E, F**). The silencing of CFTR exerted the opposite effect, leading to a substantial increase in cell invasion (**Figures 4G, H**). These observations suggest that CFTR expression is inversely associated with the motility and invasiveness of adenoid cystic carcinoma cells, which are critical steps in tumor metastasis. Furthermore, we monitored cell proliferation dynamics over a ninety six hour period using the Cell Counting Kit 8 assay. The ectopic expression of CFTR significantly impaired the growth rate of both SACC-83 and SACC-LM cells (**Figures 4I, J**). The knockdown of CFTR promoted cell proliferation, indicating a robust inhibitory effect of CFTR on tumor cell growth. The consistent suppression of migration, invasion, and proliferation upon CFTR restoration, coupled with the enhanced malignant behavior following its depletion, supports a tumor-suppressive role for CFTR in these cell lines. The loss of CFTR expression may therefore relieve intrinsic constraints on cell growth and motility, driving tumor progression and aggressive clinical behaviors.

To extend our *in vitro* observations to a physiologically relevant setting, SACC-LM cells stably expressing shCFTR and shNC were inoculated subcutaneously into the flanks of BALB/c nude mice (**Figure 4K**). Tumor volumes measured over the course of the experiment were significantly larger in the shCFTR group than in the shNC group at the indicated time points (**Figure 4L**). At the experimental end-point, tumors derived from shCFTR-expressing cells were visibly larger and weighed significantly more than control tumors (**Figure 4M**).

### CFTR expression is inversely associated with heat shock protein family members and MAPK1 in adenoid cystic carcinoma cells

The efficiency of CFTR overexpression and shRNA-mediated knockdown in SACC-83 and SACC-LM cells was first verified at both mRNA and protein levels (**Supplementary Figure 1**). We validated the transcriptomic findings using quantitative polymerase chain reaction and Western blot analyses in SACC-83 and SACC-LM cells with stable *CFTR* overexpression or knockdown.

**Figure 5:**
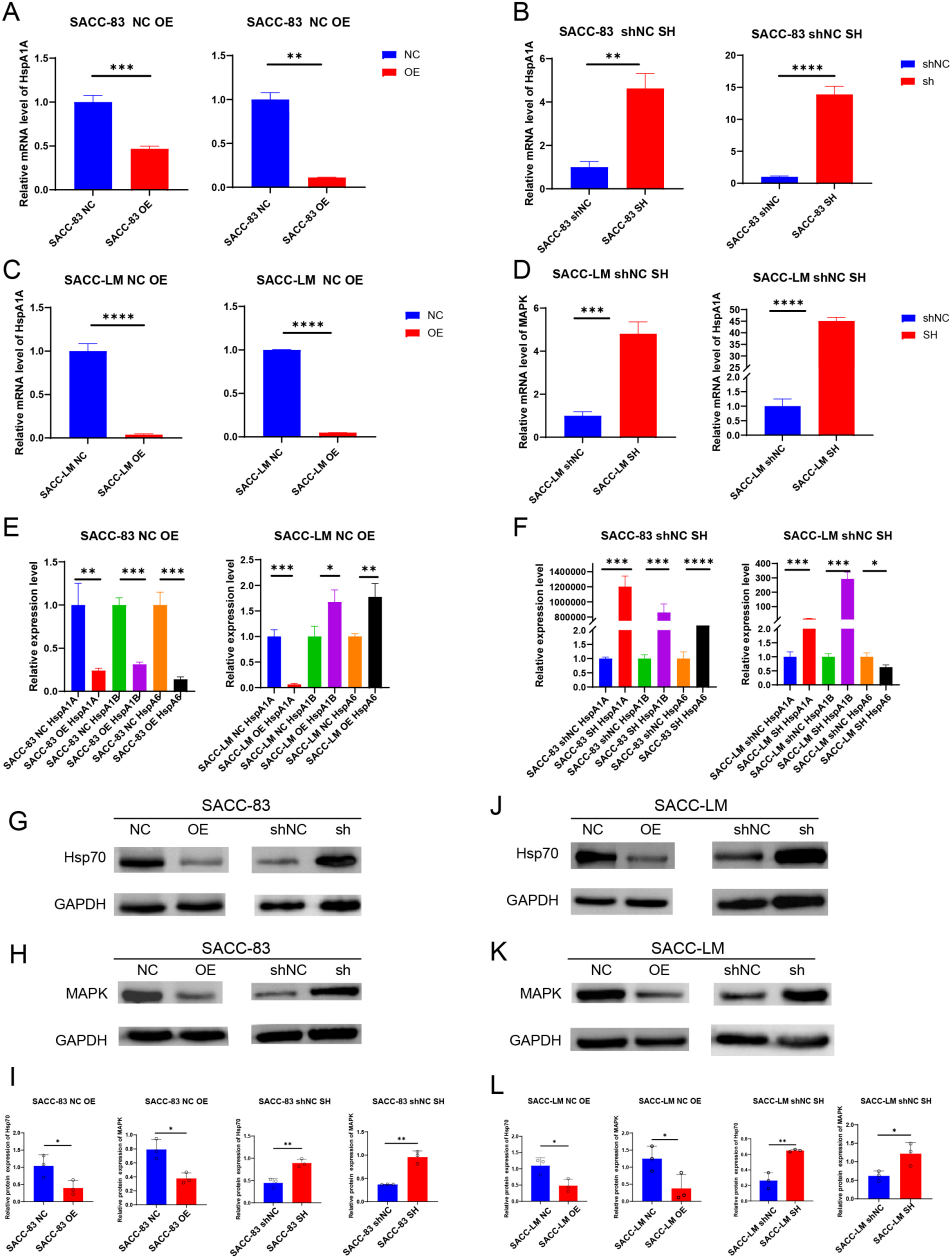
CFTR expression is inversely associated with the mRNA and protein levels of heat shock protein family members and MAPK1 in adenoid cystic carcinoma cells. **(A)** RT-qPCR analysis of HSPA1A and MAPK1 mRNA levels in SACC-83 cells following CFTR overexpression. **(B)** RT-qPCR analysis of HSPA1A and MAPK1 mRNA levels in SACC-83 cells following CFTR knockdown. **(C)** RT-qPCR analysis of HSPA1A and MAPK1 mRNA levels in SACC-LM cells following CFTR overexpression. **(D)** RT-qPCR analysis of MAPK1 and HSPA1A mRNA levels in SACC-LM cells following CFTR knockdown. **(E)** Grouped RT-qPCR comparison of HSPA1A, HSPA1B, and HSPA6 mRNA expression in SACC-83 and SACC-LM cells with CFTR overexpression. **(F)** Grouped RT-qPCR comparison of HSPA1A, HSPA1B, and HSPA6 mRNA expression in SACC-83 and SACC-LM cells with CFTR knockdown. **(G)** Representative Western blot images showing Hsp70 protein expression in SACC-83 cells with CFTR overexpression or knockdown, with GAPDH as the loading control. **(H)** Representative Western blot images showing MAPK1 protein expression in SACC-83 cells with CFTR overexpression or knockdown, with GAPDH as the loading control. **(I)** Densitometric quantification of Hsp70 and MAPK1 protein levels normalized to GAPDH in SACC-83 cells. **(J)** Representative Western blot images showing Hsp70 protein expression in SACC-LM cells with CFTR overexpression or knockdown, with GAPDH as the loading control. **(K)** Representative Western blot images showing MAPK1 protein expression in SACC-LM cells with CFTR overexpression or knockdown, with GAPDH as the loading control. **(L)** Densitometric quantification of Hsp70 and MAPK1 protein levels normalized to GAPDH in SACC-LM cells. Data are presented as mean ± SD from n = 3 independent biological experiments. *P < 0.05, **P < 0.01, ***P < 0.001, ****P < 0.0001.

At the transcriptional level, ectopic expression of CFTR in SACC-83 cells led to a significant reduction in the mRNA levels of HSPA1A and MAPK1 (ERK2, hereafter referred to as MAPK1) compared to the negative control group (**Figure 5A**). Silencing endogenous CFTR produced a pronounced upregulation of both transcripts (**Figure 5B**). This regulatory pattern was replicated in SACC-LM cells, where CFTR overexpression suppressed HSPA1A transcript abundance (**Figure 5C**) and CFTR knockdown elevated both MAPK1 and HSPA1A mRNA levels (**Figure 5D**). We quantified the mRNA expression of three heat shock protein family members, HSPA1A, HSPA1B, and HSPA6. CFTR overexpression consistently decreased the mRNA levels of all three genes in both cell lines (**Figure 5E**). CFTR knockdown elicited a robust upregulation of all three transcripts (**Figure 5F**). The reproducibility of these changes underscores the influence of CFTR on the molecular chaperone network.

Western blot analysis confirmed these transcriptional alterations at the protein level. In SACC-83 cells, Hsp70 protein abundance diminished upon *CFTR* overexpression and augmented following *CFTR* knockdown (**Figure 5G**). An identical bidirectional pattern was observed for MAPK1 protein (**Figure 5H**). Densitometric quantification confirmed the statistical significance of these differences (**Figure 5I**). These results were recapitulated in SACC-LM cells, where *CFTR* overexpression reduced Hsp70 and MAPK1 protein levels, while *CFTR* depletion elevated them (**Figures 5J–L**).

The convergence of mRNA and protein data indicates that CFTR expression is inversely associated with Hsp70 chaperone family and MAPK1 levels in SACC-83 and SACC-LM cells, although our data does not establish that CFTR directly regulates the transcription of these genes. The Hsp70 family members are cytoprotective chaperones that facilitate protein folding and suppress apoptotic cell death under proteotoxic stress. MAPK1, a key effector within the mitogen-activated protein kinase cascade, has been broadly implicated in the regulation of cell proliferation and motility. The coordinated upregulation of both pathways following CFTR loss parallels the accelerated proliferation, increased migration, and heightened invasive capacity observed in the functional assays. However, this parallel association does not establish that Hsp70 or MAPK1 causally mediate these phenotypic effects. Epistatic rescue experiments would be required to define pathway hierarchy. These data suggest that restoration of CFTR is accompanied by attenuation of the proteostasis machinery and the reduced MAPK1 protein expression, which may contribute to the re-establishment of cellular constraints on tumor progression in the SACC-83 and SACC-LM model systems.

### Pharmacological modulation of Hsp70 demonstrates its contribution to cell viability and reveals a complex relationship with MAPK1 expression in adenoid cystic carcinoma cells

We employed pharmacological strategies to directly inhibit or activate the Hsp70 pathway and assessed the consequent effects on cell viability, gene expression, and protein abundance.

**Figure 6:**
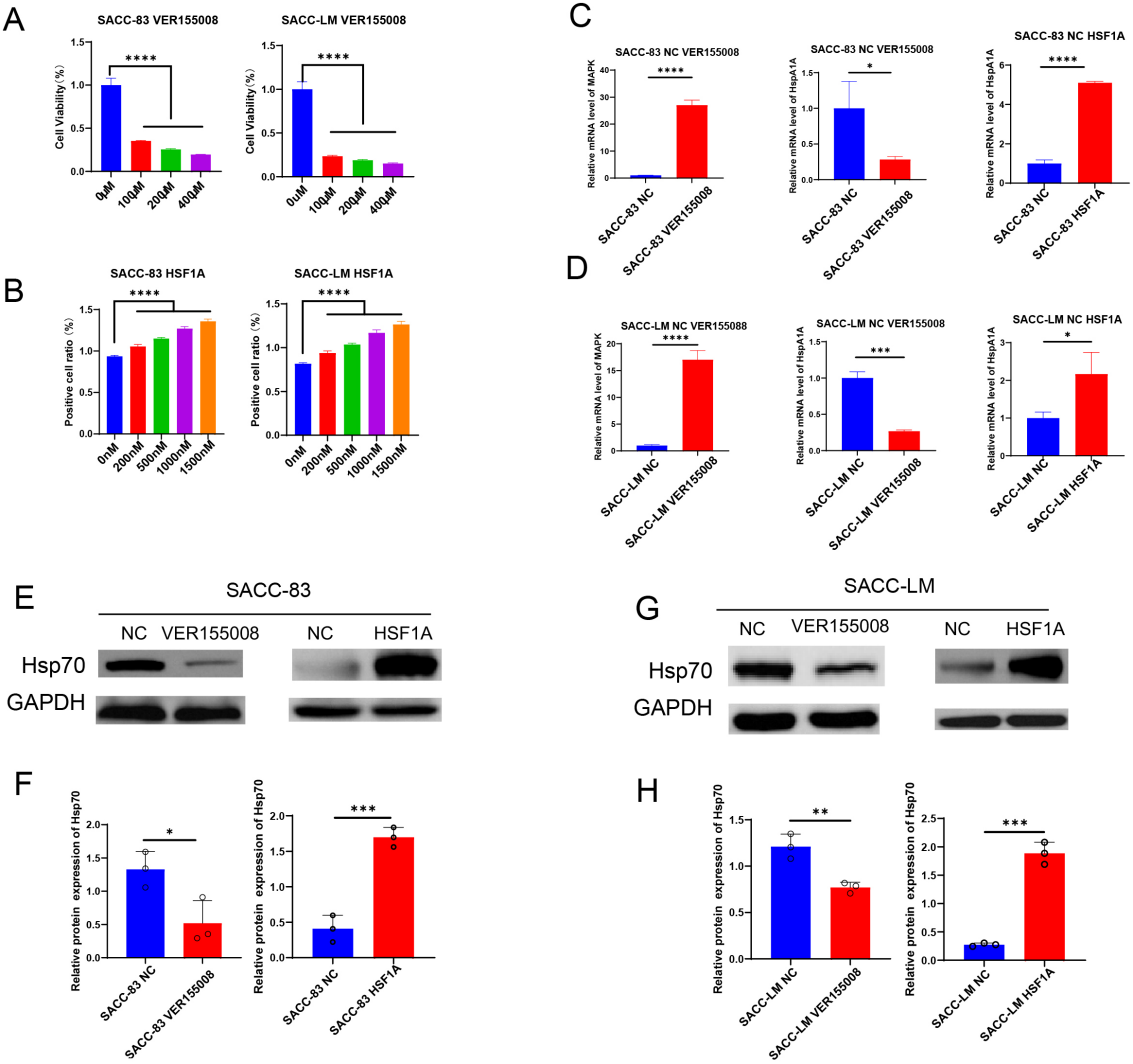
Pharmacological modulation of Hsp70 alters cell viability and reveals divergent MAPK1 transcriptional responses in SACC-83 and SACC-LM cells. **(A)** Cell viability of SACC-83 and SACC-LM cells treated with VER155008 at 0, 10, 20, and 40 μM, as determined by CCK-8 assay. **(B)** Positive cell ratio of SACC-83 and SACC-LM cells treated with HSF1A at 0, 200, 500, 1000, and 1500 nM, as determined by CCK-8 assay. **(C)** RT-qPCR analysis of MAPK1 and HSPA1A mRNA expression in SACC-83 cells following VER155008 treatment (left, middle) and HSPA1A expression following HSF1A treatment (right). **(D)** RT-qPCR analysis of MAPK1 and HSPA1A mRNA expression in SACC-LM cells following VER155008 treatment (left, middle) and HSPA1A expression following HSF1A treatment (right). **(E)** Representative Western blot images showing Hsp70 protein expression in SACC-83 cells treated with VER155008 or HSF1A, with GAPDH as the loading control. **(F)** Densitometric quantification of Hsp70 protein levels normalized to GAPDH in SACC-83 cells following VER155008 (left) and HSF1A (right) treatment. **(G)** Representative Western blot images showing Hsp70 protein expression in SACC-LM cells treated with VER155008 or HSF1A, with GAPDH as the loading control. **(H)** Densitometric quantification of Hsp70 protein levels normalized to GAPDH in SACC-LM cells following VER155008 (left) and HSF1A (right) treatment. Data are presented as mean ± SD from n = 3 independent biological experiments. *P < 0.05, **P < 0.01, ***P < 0.001, ****P < 0.0001.

Treatment with VER155008, a selective inhibitor of Hsp70, suppressed the viability of SACC-83 and SACC-LM cells in a dose-dependent manner (**Figure 6A**). Cell viability declined progressively, with the highest concentration reducing viability to approximately ten percent of the untreated control. Activation of the heat shock response through HSF1A produced a dose-dependent increase in the viable cell ratio in both cell lines (**Figure 6B**). These data suggest that Hsp70 activity is associated with the survival and proliferative capacity of SACC-83 and SACC-LM cells.

Transcriptional analysis revealed that VER155008 treatment induced an elevation of *MAPK1* mRNA in SACC-83 cells while reducing *HSPA1A* transcript levels (**Figure 6C**). HSF1A treatment drove an increase in *HSPA1A* mRNA expression. In SACC-LM cells, VER155008 upregulated *MAPK1* mRNA and elevated *HSPA1A* mRNA levels, likely reflecting a compensatory transcriptional feedback mechanism triggered by the functional blockade of Hsp70 (**Figure 6D**). The consistent upregulation of *MAPK1* transcripts following Hsp70 inhibition suggests a reciprocal regulatory relationship between the Hsp70 chaperone system and the MAPK signaling axis.

Western blot analysis corroborated these findings at the protein level. In SACC-83 cells, VER155008 treatment reduced Hsp70 protein abundance, whereas HSF1A treatment enhanced Hsp70 protein expression (**Figures 6E, F**). An analogous pattern was observed in SACC-LM cells (**Figures 6G, H**).

These pharmacological experiments provide independent support for the functional importance of Hsp70 in sustaining the malignant phenotype of adenoid cystic carcinoma cells. Pharmacological inhibition of Hsp70 by VER155008 suppressed cell viability in a manner consistent with the anti-proliferative effects observed following CFTR restoration, supporting the notion that elevated Hsp70 activity contributes to tumor cell survival in the CFTR-deficient state. It should be noted, however, that VER155008 broadly disrupts proteostasis and induces apoptosis, and that the observed reductions in proliferation, migration, and invasion may therefore reflect generalized cellular stress in addition to, or rather than, selective targeting of a CFTR-specific vulnerability. Similarly, HSF1A-mediated activation of Hsp70 promoted cell viability, paralleling the proliferative advantage conferred by CFTR loss. However, an important divergence was observed at the level of MAPK1 regulation. Whereas CFTR overexpression coordinately suppressed both Hsp70 and MAPK1 expression (**Figure 5**), pharmacological Hsp70 inhibition was accompanied by a pronounced transcriptional upregulation of MAPK1 (**Figures 6C, D**), an effect opposite to that of CFTR restoration. This discordance indicates that CFTR does not regulate MAPK1 solely or primarily through Hsp70, and instead suggests that CFTR is inversely associated with Hsp70 and MAPK1 through parallel, at least partially independent, mechanisms. The compensatory upregulation of MAPK1 transcripts following Hsp70 blockade may reflect a stress-adaptive transcriptional response to the disruption of chaperone-dependent protein homeostasis. Despite this molecular divergence, the convergence of VER155008 treatment and CFTR restoration on the reduction of proliferation, migration, and invasion suggests that Hsp70 inhibition attenuated malignant phenotypes in these model systems, likely reflecting the cytoprotective and anti-apoptotic functions of the Hsp70 chaperone system. The sensitivity of SACC-83 and SACC-LM cells to VER155008 indicates that pharmacological targeting of Hsp70 warrants further evaluation as a strategy for restraining tumor progression in cases with diminished CFTR expression.

### Pharmacological modulation of heat shock protein 70 alters the migration and invasion of adenoid cystic carcinoma cells

We employed pharmacological strategies to determine whether the functional status of heat shock protein 70 influences the motility and invasiveness of adenoid cystic carcinoma cells.

**Figure 7:**
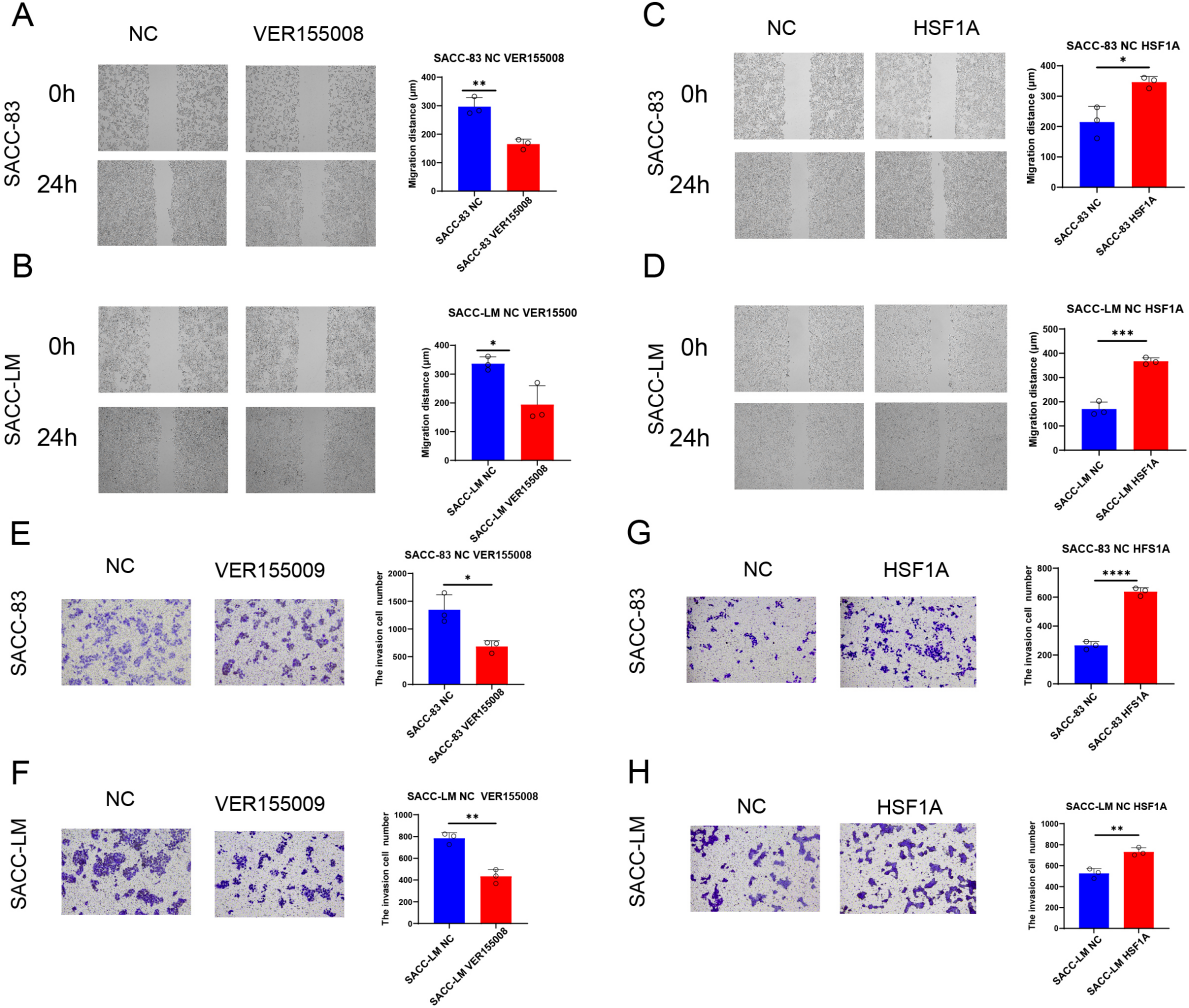
Pharmacological modulation of Hsp70 alters the migration and invasion capacities of SACC-83 and SACC-LM cells. **(A, B)** Wound-healing assays evaluating the migration of SACC-83 **(A)** and SACC-LM **(B)** cells following VER155008 treatment. **(C, D)** Wound-healing assays evaluating the migration of SACC-83 **(C)** and SACC-LM **(D)** cells following HSF1A treatment. **(E, F)** Transwell invasion assays assessing the invasive capacity of SACC-83 **(E)** and SACC-LM **(F)** cells treated with VER155008. **(G, H)** Transwell invasion assays assessing the invasive capacity of SACC-83 **(G)** and SACC-LM **(H)** cells treated with HSF1A. Data are presented as mean ± SD from n = 3 independent biological experiments. *P < 0.05, **P < 0.01, ***P < 0.001, ****P < 0.0001.

Treatment with the inhibitor VER155008 substantially impaired the migratory properties of the tumor cells. SACC-83 and SACC-LM cells subjected to VER155008 exhibited a significantly reduced migration distance in the wound healing assay (**Figures 7A, B**). Pharmacological activation of the heat shock response via HSF1A accelerated wound closure and increased migration distance in both cell lines (**Figures 7C, D**). Transwell invasion assays corroborated these functional shifts. The blockade of Hsp70 activity severely restricted the ability of the cells to traverse the Matrigel barrier, leading to a decrease in the number of invading cells (**Figures 7E, F**). HSF1A-mediated upregulation of Hsp70 function augmented the invasive capacity of both cell lines (**Figures 7G, H**).

The observed effects of Hsp70 modulation on cell migration and invasion are consistent with its known role in stabilizing client proteins involved in focal adhesion turnover and extracellular matrix degradation. However, because VER155008 impairs cell viability and induces apoptosis, the reductions in wound closure and invading cell numbers observed following Hsp70 inhibition should be interpreted as reflecting the combined consequences of impaired proliferation, survival, and motility rather than a selective anti-migratory mechanism. Dedicated experiments that uncouple motility from cell death, such as mitomycin C-arrested wound healing or single-cell tracking under sublethal dosing conditions, would be required to determine whether Hsp70 inhibition exerts a cell-autonomous anti-migratory effect. The enhancement of migration and invasion upon HSF1A-mediated Hsp70 activation provides complementary evidence that the functional status of this chaperone system influences the aggressive phenotype of SACC-83 and SACC-LM cells. The sensitivity of these tumor cells to Hsp70 blockade highlights the therapeutic rationale for targeting this pathway in ACC, while acknowledging that the contribution of cytotoxicity to the observed anti-migratory effects requires further clarification.

### Pharmacological and genetic modulation of heat shock protein 70 modulates the proliferation and survival of adenoid cystic carcinoma cells

We conducted proliferation and apoptosis assays using pharmacological agents and genetic manipulation to elucidate the contribution of heat shock protein 70 to tumor cell expansion and survival.

**Figure 8:**
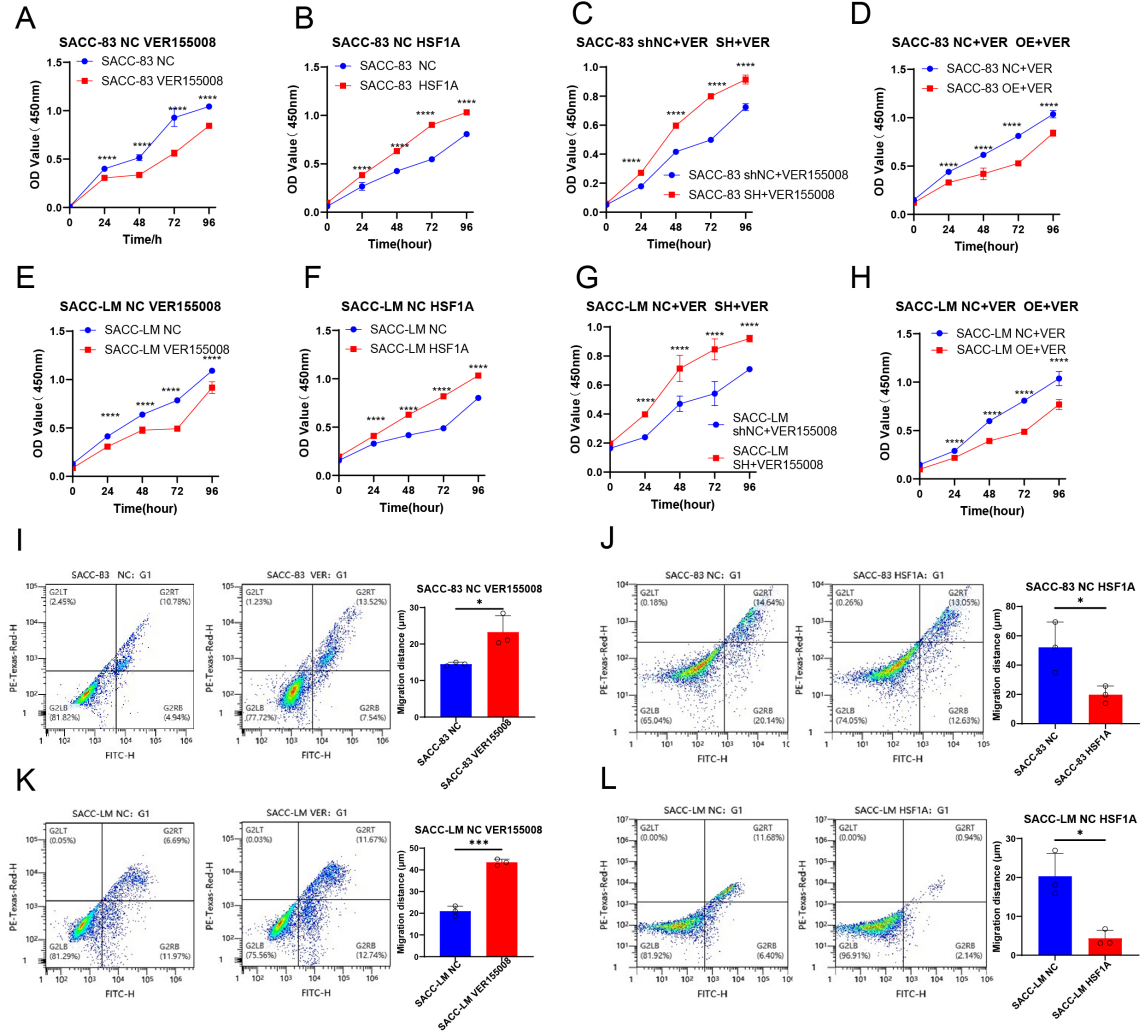
Pharmacological and genetic modulation of Hsp70 modulates the proliferation and apoptosis of SACC-83 and SACC-LM cells. **(A, B)** CCK-8 assays evaluating the proliferation of SACC-83 cells following VER155008 **(A)** or HSF1A **(B)** treatment. **(C, D)** CCK-8 assays evaluating the proliferation of SACC-83 cells with Hsp70 knockdown **(C)** or overexpression **(D)** combined with VER155008 treatment. **(E, F)** CCK-8 assays evaluating the proliferation of SACC-LM cells following VER155008 **(E)** or HSF1A **(F)** treatment. **(G, H)** CCK-8 assays evaluating the proliferation of SACC-LM cells with Hsp70 knockdown **(G)** or overexpression **(H)** combined with VER155008 treatment. **(I, J)** Flow cytometry analysis of apoptosis in SACC-83 cells treated with VER155008 **(I)** or HSF1A **(J)**. **(K, L)** Flow cytometry analysis of apoptosis in SACC-LM cells treated with VER155008 **(K)** or HSF1A **(L)**. Data are presented as mean ± SD from n = 3 independent biological experiments. *P < 0.05, ****P < 0.0001.

The application of the Hsp70 inhibitor VER155008 suppressed the proliferative trajectory of the tumor cells. VER155008 treatment resulted in a significant reduction in cell growth over a ninety-six-hour period in both SACC-83 and SACC-LM cells (**Figures 8A, B**). Activation of the heat shock response via HSF1A accelerated cell proliferation and led to elevated growth curves. When the inhibitor was applied to cells with altered Hsp70 expression levels, Hsp70 knockdown cells exhibited a higher proliferative rate than the control knockdown cells subjected to the same treatment (**Figures 8C, G**). Hsp70 overexpressing cells demonstrated increased sensitivity to the inhibitor and showed reduced proliferation compared to the control group (**Figures 8D, H**).

Flow cytometric analysis revealed that pharmacological inhibition of Hsp70 by VER155008 robustly induced apoptosis in SACC-83 and SACC-LM cells (**Figures 8I, K**). Treatment with the activator HSF1A conferred a survival advantage and reduced the baseline apoptotic rates in both cell lines (**Figures 8J, L**). These dynamic shifts in proliferation and apoptosis underscore the function of Hsp70 as a central node in the cellular survival network. The molecular chaperone buffers proteotoxic stress and stabilizes anti-apoptotic proteins to facilitate continuous tumor growth. The differential responses observed in the genetically modified cells upon inhibitor treatment suggest complex compensatory mechanisms or altered reliance on the chaperone machinery. The induction of apoptosis following Hsp70 blockade highlights the vulnerability of SACC-83 and SACC-LM cells to the disruption of their protein homeostasis network and reinforces the therapeutic rationale for targeting this chaperone system.

## Discussion

In this study, we identified CFTR as a candidate tumor suppressor and a potential prognostic indicator in adenoid cystic carcinoma. Transcriptomic analyses across multiple independent cohorts revealed the consistent downregulation of CFTR, a molecular event that is associated with inferior patient survival. Functional investigations in the SACC-83 and SACC-LM cell lines demonstrated that modulation of CFTR expression consistently altered tumor cell proliferation, migration, and invasion *in vitro*, supporting a tumor-suppressive role for this gene in these model systems. Mechanistically, CFTR deficiency is associated with the dysregulation of epithelial proteostasis, the upregulation of the Hsp70 chaperone network, and elevated MAPK1 protein expression. These molecular alterations may collectively contribute to enhanced tumor cell survival and motility, although the extent to which they individually drive malignant progression remains to be dissected, and formal epistatic experiments establishing causal mediation are lacking.

The prominent transcriptional suppression of *CFTR* in adenoid cystic carcinoma aligns with its recognized role as a tumor-suppressive channel in diverse epithelial malignancies. Previous investigations have documented the epigenetic silencing and transcriptional repression of *CFTR* in colorectal, breast, and lung cancers, where its deficiency accelerates disease progression (8). Our cross-cohort analysis indicates that CFTR suppression is a recurrent and reproducible transcriptional feature of adenoid cystic carcinoma rather than a stochastic alteration. The early and sustained divergence in survival curves between patients with varying CFTR levels suggests that CFTR expression is associated with the long-term clinical trajectory of the disease. The loss of CFTR may compromise the differentiated secretory phenotype of the salivary epithelium, potentially contributing to a phenotypic shift toward a less differentiated and more proliferative state (24).

It is important to position CFTR within the established molecular framework of ACC. The MYB-NFIB fusion oncogene represents the most well-characterized genetic driver in ACC, and its downstream transcriptional program is thought to sustain the undifferentiated proliferative state of tumor cells. In addition, recurrent alterations in NOTCH, FGF, and PI3K pathway components have been implicated in ACC progression and represent emerging therapeutic targets. Our study identifies CFTR as a novel element within this molecular landscape, and we propose that CFTR silencing may cooperate with or operate independently of these established pathways. Specifically, the proteostatic vulnerability that accompanies CFTR loss may represent a metabolic dimension of ACC pathobiology that complements the genomic and transcriptional programs driven by MYB-NFIB and other ACC-associated alterations. However, direct experimental investigation of the relationship between CFTR status and MYB-NFIB activity, NOTCH signaling, or other ACC-defining pathways was beyond the scope of the present study and remains an important direction for future research (31).

Network topology and functional enrichment analyses position CFTR as a highly connected node within the downregulated gene network. Enrichment of cyclic AMP and protein kinase A-related terms among CFTR interactors suggests a potential involvement of this pathway, although direct experimental evidence for its activation or disruption in adenoid cystic carcinoma was not obtained in this study. The predicted interaction between CFTR and the protein kinase A catalytic subunits PRKACA and PRKACB, derived from protein interaction databases, raises the possibility that CFTR may contribute to the spatial organization of localized kinase signaling, a hypothesis that warrants direct biochemical investigation. Because we have not performed co-immunoprecipitation, proximity ligation or direct measurement of cAMP/PKA activity levels in ACC cells, the CFTR–cAMP/PKA link reported in our study should be regarded as a bioinformatically generated hypothesis rather than an established mechanism. Future studies employing cAMP biosensors or PKA activity reporters in ACC cells will be needed to evaluate this hypothesis experimentally. The downregulation of CFTR is accompanied by broad transcriptional reprogramming consistent with altered cellular metabolism. We observed a marked enrichment of ribosome biogenesis and spliceosome pathways in CFTR-deficient tumors, representing a canonical metabolic adaptation in rapidly dividing neoplastic cells (25). This metabolic and transcriptional reprogramming is reminiscent of patterns reported in other glandular neoplasms following the disruption of secretory differentiation programs, although whether cyclic AMP-dependent growth inhibition is specifically involved in adenoid cystic carcinoma requires further investigation (26).

The data suggest a functional relationship between CFTR and components of the cellular proteostasis machinery. RNA sequencing following transient CFTR overexpression revealed a coordinated transcriptional suppression of multiple heat shock protein family members, including HSPA1A, HSPA1B, HSPA6, HSPH1, and DNAJB1, with enrichment of proteostasis and endoplasmic reticulum stress pathways among the downregulated genes (27). This finding was corroborated by stable CFTR restoration, which produced a sustained reduction in the expression of Hsp70 family members (HSPA1A, HSPA1B, HSPA6) and MAPK1 at both the mRNA and protein levels. The convergence of these results from independent experimental paradigms indicates that CFTR expression is inversely associated with the activity of the cellular chaperone network. The pharmacological interventions performed in this study provide further support for a functional role of the Hsp70 chaperone network in sustaining the malignant phenotype of adenoid cystic carcinoma cells. Targeted inhibition of Hsp70 utilizing VER155008 robustly attenuated cell migration, invasion, and proliferation while simultaneously triggering apoptotic cell death. These phenotypic consequences parallel the tumor-suppressive effects observed upon stable CFTR restoration, suggesting functional convergence between the two perturbations. An apparent discrepancy merits explicit discussion. CFTR overexpression coordinately suppressed both Hsp70 and MAPK1 at the mRNA and protein levels (**Figure 5**), whereas pharmacological Hsp70 inhibition by VER155008 reduced Hsp70 protein but elicited a pronounced compensatory upregulation of MAPK1 mRNA (**Figures 6C, D**). We interpret this divergence as evidence against a strictly linear CFTR–Hsp70–MAPK1 cascade and in favor of parallel regulation, whereby CFTR is associated with both arms upstream. Pharmacological blockade of Hsp70 disrupts chaperone-dependent proteostasis and activates stress-adaptive transcriptional programmes, including HSF1 and stress-kinase signaling, that can compensatorily induce mitogenic effectors such as MAPK1 as a survival response, consistent with published observations that proteotoxic stress engages compensatory mitogenic programmes. Despite this MAPK1 induction, the anti-proliferative, anti-migratory and pro-apoptotic effects of VER155008 were maintained, indicating that the cytoprotective function of Hsp70 is dominant over the compensatory MAPK1 response in ACC cells. Direct epistatic validation of this parallel-regulation model by Hsp70 re-expression in CFTR-restored cells and by MAPK1 inhibition in CFTR-deficient cells is required and represents a priority for future work. The compensatory MAPK1 transcriptional response to Hsp70 blockade may reflect a stress-adaptive feedback loop, consistent with reports that proteotoxic stress can activate mitogenic signaling as a survival response (28, 29). Notably, despite this MAPK1 upregulation, VER155008 treatment effectively suppressed proliferation, migration, and invasion and induced apoptosis, indicating that the loss of Hsp70 cytoprotective function is dominant over the compensatory MAPK1 induction. These observations suggest that CFTR loss is associated with increased cellular reliance on Hsp70 for proteostatic buffering and on MAPK1 for mitogenic drive, and that pharmacological disruption of either arm may attenuate the malignant phenotype in the SACC-83 and SACC-LM model systems, although the two arms appear to be regulated independently rather than in a hierarchical cascade. It should be emphasized that our study does not formally demonstrate selective addiction or synthetic vulnerability linked specifically to CFTR deficiency, and the term “vulnerability” is used to describe a potentially exploitable biological state rather than an established therapeutic dependency. Several limitations should be acknowledged. First, our functional findings are based on a single ACC cell lineage, the SACC-83 and SACC-LM pair. SACC-83 and SACC-LM are closely related sublines that share the same patient-of-origin, and they should therefore be regarded as a single biological system rather than truly independent ACC models. Validation in additional genetically independent ACC cell lines, primary cultures, or organoids is needed to assess the generalizability of the CFTR-Hsp70 axis. Second, the survival association was derived from a public transcriptomic cohort (n = 29; 20 death events) and, while providing statistical power for the log-rank test, yields wide confidence intervals that limit the precision of the hazard ratio estimate. Protein-level confirmation together with multivariable analysis in an independent, fully annotated tissue cohort is required before firm prognostic conclusions can be drawn. Third, although functional phenocopying between CFTR restoration and Hsp70 inhibition was observed, whether Hsp70 upregulation is necessary for the phenotype driven by CFTR loss and whether MAPK1 is a causal mediator remain to be determined by epistatic rescue experiments, including Hsp70 re-expression in CFTR-restored cells and MAPK1 inhibition in CFTR-deficient cells. Because the Hsp70 inhibitor VER155008 impairs viability and induces apoptosis, the reduced wound closure and invasion may partly stem from cytotoxicity. Dedicated assays that uncouple motility from cell death, such as mitomycin C-arrested wound healing or single-cell tracking under sublethal conditions, are required to demonstrate a cell-autonomous anti-migratory effect. Although xenograft data confirm a tumor-suppressive role for CFTR, comprehensive *in vivo* pharmacological profiling of Hsp70 inhibitors, including efficacy, pharmacokinetics, on-target specificity and systemic toxicity, has not been undertaken. Lastly, the proposed CFTR-cAMP/PKA connection relies exclusively on PPI-network modeling and pathway enrichment without direct biochemical evidence in ACC cells and should be regarded as a bioinformatically generated hypothesis. Confirmation of these results in additional genetically distinct ACC cell lines or patient-derived primary cultures would strengthen the generalizability of the conclusions. Nevertheless, the absence of complex *in vivo* metastasis models and of a dedicated pharmacological arm for Hsp70 inhibition restricts the ability to evaluate the systemic efficacy, pharmacokinetics, on-target specificity and toxicity of Hsp70 inhibition in ACC. Future research should incorporate patient-derived xenografts and orthotopic animal models to evaluate the therapeutic efficacy and systemic toxicity of targeting the CFTR-Hsp70 axis in a more physiologically relevant context (30).

These findings collectively support a role for CFTR as a candidate prognostic biomarker and functional tumor suppressor in adenoid cystic carcinoma, with the caveat that functional conclusions are primarily supported within the SACC-83 and SACC-LM model system. The pathological silencing of CFTR is associated with increased cellular reliance on the Hsp70 chaperone system and elevated MAPK1 expression that may sustain malignant growth. Whether this vulnerability arises from the disruption of cyclic AMP-dependent regulatory mechanisms or from alternative downstream consequences of CFTR loss remains an important question for future studies. In the SACC-83/SACC-LM lineage, pharmacological blockade of Hsp70 attenuated the proliferative and migratory phenotypes observed in CFTR-deficient adenoid cystic carcinoma cells *in vitro*, and shRNA-mediated knockdown of CFTR accelerated xenograft growth *in vivo*, thereby corroborating the tumor-suppressive role of CFTR. Taken together, these findings provide a preclinical rationale for further investigation of proteostasis inhibitors as a candidate therapeutic strategy for patients with CFTR-deficient adenoid cystic carcinoma. Dedicated *in vivo* pharmacological studies addressing efficacy, pharmacokinetics, on-target specificity and systemic toxicity of Hsp70 inhibitors in ACC xenograft and patient-derived models will be required before clinical translation can be contemplated. Furthermore, validation of these results in genetically independent ACC cell lines, patient-derived primary cultures, or organoid models is essential to establish the generalizability of the CFTR-Hsp70 axis beyond the SACC-83/SACC-LM system.

## Data Availability

The data presented in this study are deposited in the OMIX repository, China National Center for Bioinformation (CNCB), under accession number OMIX017879 (https://ngdc.cncb.ac.cn/omix/).
