# Supplementary material for: CFTR functions as a tumor suppressor in adenoid cystic carcinoma and its silencing reveals an associated vulnerability involving the Hsp70 chaperone system
Source: Front Oncol. 2026 Jul 6;16:1836263. doi: 10.3389/fonc.2026.1836263 (PMC13381213; doi:10.3389/fonc.2026.1836263)
Supplement: Supplementary file 1 [file DataSheet1.pdf]

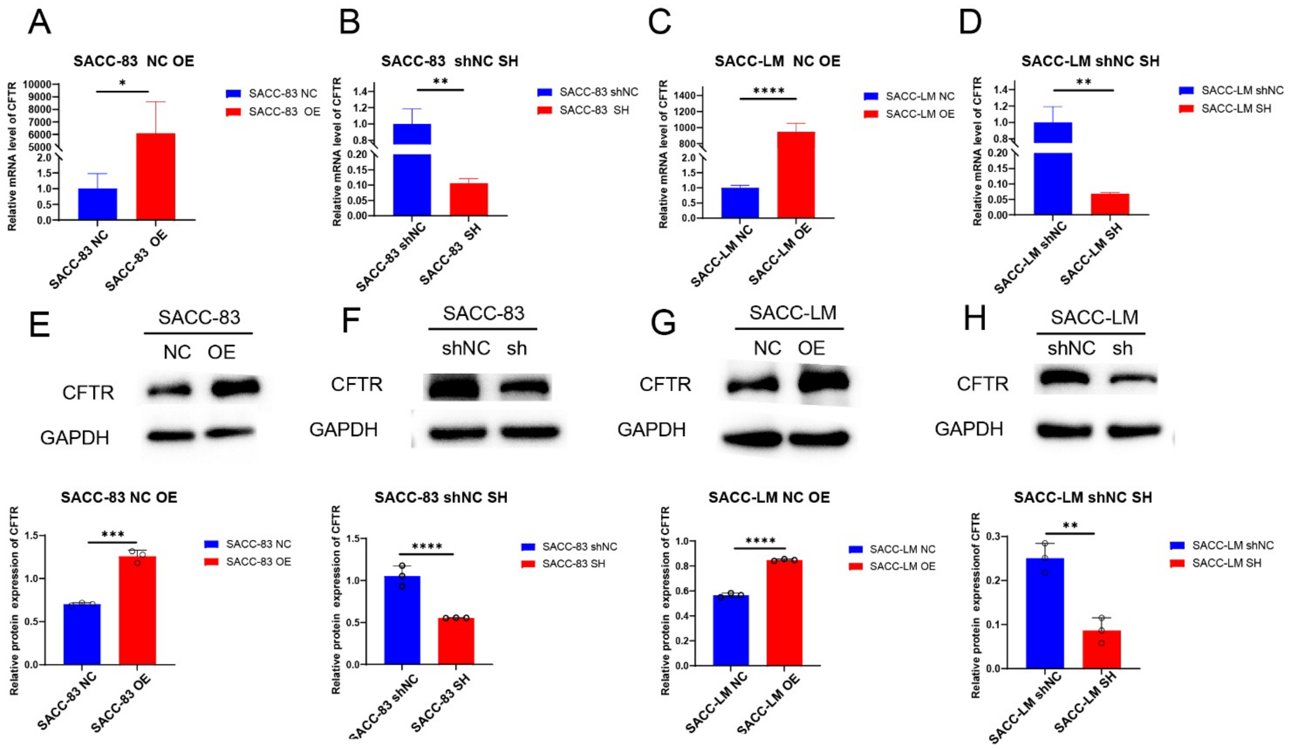

Supplementary Figure S1. Validation of CFTR overexpression and shRNA-mediated knockdown efficiency in SACC-83 and SACC-LM cells. (A) Relative CFTR mRNA levels in SACC-83 cells transfected with empty-vector control (NC) or CFTR-overexpression construct (OE), quantified by RT-qPCR and normalised to GAPDH. (B) Relative CFTR mRNA levels in SACC-83 cells transfected with non-targeting shRNA control (shNC) or CFTR-targeting shRNA (SH). (C) Relative CFTR mRNA levels in SACC-LM cells transfected with NC or OE constructs. (D) Relative CFTR mRNA levels in SACC-LM cells transfected with shNC or SH constructs. (E–H) Representative Western blot images (upper panels) and densitometric quantification (lower panels) of CFTR protein expression, with GAPDH as the loading control, in SACC-83 NC vs OE (E), SACC-83 shNC vs sh (F), SACC-LM NC vs OE (G) and SACC-LM shNC vs sh (H) cells. Data are presented as mean ± SD from n = 3 independent biological experiments; statistical significance was determined by unpaired two-tailed Student's t-test. *P < 0.05, **P < 0.01, ***P < 0.001, ****P < 0.0001.
